# Corporate Engagement Strategies in Northern Mining: Boliden, Sweden and Cameco, Canada

**DOI:** 10.1007/s00267-023-01854-5

**Published:** 2023-07-27

**Authors:** Gregory Poelzer

**Affiliations:** Luleå University of Technology, Political Science, Luleå, Sweden

**Keywords:** CSR, Governance, Social license to operate, Mining, Indigenous communities

## Abstract

The role of corporations in societal outcomes continues to grow. Mining companies now face the expectation of not only providing economic benefits to communities, but act as a facilitator for social wellbeing and environmental stewardship. In the mining sector, this has placed renewed attention to defining corporate social responsibility and, in turn, how social license to operate is understood. These developments are particularly pertinent when mining operations affect Indigenous communities – where land use is central to livelihood. This study looks at the community engagement strategies of two mining companies in northern countries, Cameco (Canada) and Boliden (Sweden). By comparing their approaches, this paper explores the development of their practices over time and assess to what extent their corporate policy has translated into everyday practice and outcomes. The findings of demonstrate that high levels of trust are established when corporate approaches are built around transparency and collaboration – resulting in agreements that include long-term partnerships around socio-economic and environmental management.

## Introduction

### Corporate Approach in the Minerals Industry

Relationships between mining companies and communities are a defining characteristic in successful operations. Social license to operate (SLO) has become the dominant concept in understanding the perception of mining companies, stemming from the need to address bad corporate practices in the global south and mitigate risk for the mining sector (Meesters et al. 2021). However, the rise in commodity prices and the resultant proliferation of the mining sector expanded scrutiny to nearly all on-going and planned mines. Shifting the focus not only on the symptoms of bad relationships between companies and communities but also on the solutions that foster positive ones, many of which come from the global north. Further, relationships between mining companies and Indigenous communities are particularly sensitive due to competing use of land and, as a result, there has been a global shift towards recognizing the rights of communities specifically Indigenous, regarding extractive activities on their traditional territories (Anaya 2005; Tomlinson 2019; Åhrén 2016). To investigate different approaches used to facilitate trust with Indigenous communities, this paper looks at the strategies employed by two companies operating mines in northern regions: Boliden’s Aitik operations in Norrbotten, Sweden and Cameco’s McArthur River/Key Lake operations in Saskatchewan, Canada.

Gaining acceptance of communities in proximity of mining operations is one of the primary goals of mining companies today. SLO at the community level continues to be the predominant conceptualization of the term (Prno 2013; Martinez and Franks 2013; Koivurova et al. 2015; Wilson 2016). It has been modeled and studied in many different geographic, cultural and governance contexts (Thomson and Boutilier 2011; Moffat and Zhang 2014; Mercer-Mapstone et al. 2017; Lesser et al. 2021). SLO is now central to many discussions around mineral development because meeting the legislative requirements is no longer a guarantee for success in mineral extraction (Prno 2013). In both the Canadian and Swedish mining contexts, Indigenous communities are one of the most prominent actors involved in the governance of mineral resource development, particularly given the importance of land use, and are often conceptualized as the ‘community’ component of the government-company-community relationship. Importantly, despite all three actors bringing different expectations to a project, positive outcomes are possible when consensus and trust between parties is reached (Moffat et al. 2016) via well-defined measures (Zhang et al. 2015).

The cases of Boliden and Cameco offer examples of companies that operate mines with little contestation. However, Cameco currently enjoys a strong SLO while Boliden possesses a weak SLO, which provides an interesting comparison for looking at difference corporate approaches, guided by the space to maneuver within the legislative framework. This overall aim of this article is to compare approaches used by mining companies to engage with Indigenous communities and assess their ability to develop a SLO.

## Theory

### Corporate engagement, SLO, and community

The nature of corporate engagement has developed significantly over the past decades. As the corporate world began to recognize the influence of actors beyond investors, due in large part to environmental regulation, focus on the identifying different organizations, associations, or groups that could affect operations took root (Freeman 1984). Throughout the resource development sector, corporate social responsibility (CSR) is one important mechanism to address stakeholder concerns. CSR largely includes activities that go beyond core corporate activities and legally required behavior, addressing stakeholders’ environmental and social concerns (Trebeck 2017). CSR is premised on the idea that corporations have the capacity to conduct their affairs in a manner that affects the well-being of potentially impacted communities. This means that mining companies often address such matters as environment management practice, social and community development, local employment and labor, and human rights (Campbell 2011). The extent to which these activities meet the expectations of communities is often referred to as social license to operate.

SLO is the ongoing acceptance or approval of an operation by the stakeholders who are affected by it (Joyce and Thomson 2000; Nelsen and Scoble 2006; Thomson and Joyce 2008; Thomson and Boutilier 2011; Moffat and Zhang 2014) and, like CSR, places particular focus on stakeholders who can affect its profitability (Graafland 2002). For industries involved in resource development, embracing sustainability and sustainable development has meant working more closely with stakeholders to maintain a SLO (Solomon et al. 2008; Prno and Slocombe 2012). Therefore, obtaining SLO has nearly become essential for extractive industries as key stakeholders increasingly expect the industry to contribute positively to the community and to communicate openly and engage the local communities in their decision-making (Moffat and Zhang 2014).

Previous work on SLO in northern mining nations analyzed how a community responded to the behavior of mining companies. One study looked at mines located in Norway, Finland, Russia, and Sweden where they found that the approach taken by companies affected the legitimacy or credibility of operations while also recognizing the effect of contextual factors (Koivurova et al. 2015). These cases demonstrated that community engagement needs to be persistent and that fostering acceptance from a community is an on-going process. Another example, where social license was established comes from the Red Dog (zinc-lead) mine in Alaska, USA (Prno 2013). Here support from community members in the region was linked to mine’s operation importance in the local economy. The community viewed the mine as a fair distributor of financial benefit and ensured community members’ participation in decision-making processes (Prno 2013). Community engagement, therefore, is central to recognizing the interests of Indigenous communities and focus on the impact of extractive activities on traditional Indigenous territories has become increasingly important (Anaya 2005; Tomlinson 2019; Åhrén 2016). However, considerable literature demonstrates that in many cases, government and industry continue to fail to consult with affected communities adequately and find collaborative solutions related to the exploitation of natural resources (Anaya 2004; Hanna and Vanclay 2013: Tomlinson 2019). Thus, attention is needed to examine cases where the relationship between mining companies and Indigenous communities appears to function.

### SLO and Governance

Acceptance throughout the duration of a mining project requires the recognition, understanding, and response to different sets of expectations. Because the extraction of natural resources elicits a spectrum of perspectives, ranging from support to dissent, the task of gaining SLO becomes more complex through the involvement of more actors and creation of new relationships. These relationships are particularly critical with Indigenous communities where decision on land use hold long-term implications. Governance can be understood as how government, industry, and communities organize themselves to make important decisions regarding the use and protection of their common resources (Armitage et al. 2018). Some scholars perceive governance as an interactive process of steering the affairs of both state and non-state actors (Kooiman 1993; Jessop 2002), which includes the formulation and application of principles guiding those interactions (Kooiman et al. 2005). The broader transition from government to governance has yielded a broadened range of governing actors in mining projects, resulting in industry and civil society sharing governing responsibilities with governments (Ballard and Banks 2003; Lemos and Agrawal 2006; McAllister and Fitzpatrick 2010; Prno and Slocombe 2012). Government does not decide alone but needs non-governmental actors and stakeholders to contribute on issues of resource use and sustainable development – in this case the focus is placed on governance between mining companies and Indigenous communities.

Substantial stakeholder involvement from the start of the project is crucial because SLO failures have mainly been due to a lack of engagement in the governance of a resource (Smits et al. 2016). The concept of governance is a step towards a more dynamic field of engagement between government, civil society and industries increasing the participation in the processes as it is often this participation that results in either community acceptance or rejection of a project (Dredge and Whitford 2011; Hall 2011). The shift to involve stakeholders is especially relevant to the emergence of SLO as there are an increased level of demand for new input in decision-making (McMahon and Remy 2001). Moreover, the relevance of stakeholders such as community to the work of government, and industry has increased over the years (Parmar et al. 2010); their criticism significantly affects industries’ actions (Barnett 2007), as they seek to legitimize their operations, and this requires connecting with stakeholders and meeting their needs (Chen and Roberts 2010).

Economic development versus environmental conservation, national interest versus local benefits, and societal transformation versus cultural preservation all serve as points of divergence amongst actors involved in and affected by mining development (Bebbington and Williams 2008; Anguelovski 2011; Arellano-Yanguas 2011). Normatively, the compatibility of mining with sustainability continues to drive much of the debate – whether it can adapt to novel demands and remain a staple of the future economy (McMahon and Remy 2001). Mining demands the recognition of various interests, in this case Indigenous, to move projects forward. In cases with significant divergence on views of a sustainable future, open contestation can occur. From orderly objections raised during public consultations to blockades and mass protest, negative reactions to mining take many forms and, in some cases, hold important repercussions for the viability of the project.

### Company to Community Governance

To reduce the potential for contestation, governments attempt to enhance the policy process (or elements of it) with more inclusivity while mining companies emphasize corporate social responsibility (CSR) in their efforts to improve community relations (McMahon and Remy 2001). While much of the research on corporate engagement is focused on developing countries, where the legislative and regulatory are typically lacking, the contributions here add to our understanding on how corporate practices look within a strong institutional framework (Frederiksen 2019) and contribute to trust building with the community (Cesar and Jhony 2020) or, alternatively, fail to deliver on its promise and perpetuate historical problems (Hilson et al. 2019). To that end, one of the most important aspects of CSR and mining today is the impacts for Indigenous communities, particularly the potential economic benefits that come with company-community partnerships (Berman et al. 2020). The connection between CSR and partnerships is particularly pertinent to Sweden and Canada, which are both home to Indigenous populations. Again, while the importance of these engagement and feedback mechanisms for securing local benefits is acute in developing states with weak institutions, these issues remain pertinent for jurisdictions that are engaged in debates over the benefits and costs of mineral extraction. Often regarded as progressive countries in terms of environmental stewardship and human welfare, studying these examples demonstrates the extent to which mining companies contribute to societal goals.

To obtain SLO with communities, some make the argument for early communication; transparent disclosure of information; development of conflict resolution mechanisms; and culturally appropriate decision-making (Goldstuck and Hughes 2010). To regulate these types of activities, formal agreements are reached between mining companies and communities. These agreements, often in the form of Impact Benefit Agreements (IBAs), contain mechanisms that, on the benefit-side, provide opportunities for the community brought by mining development and, on the impact-side, address adverse socio-economic and environmental impacts. Also included are procedures around communication, reporting, and accountability measures. At the core of these agreements is the aim to achieve “a more sustainable mining development by…engaging in the appropriate level of consultation and providing adequate benefits and compensation” (Hitch and Fidler 2007). In these types of arrangements, we see political outcomes achieved through company-community collaboration.

**Fig. 1:**
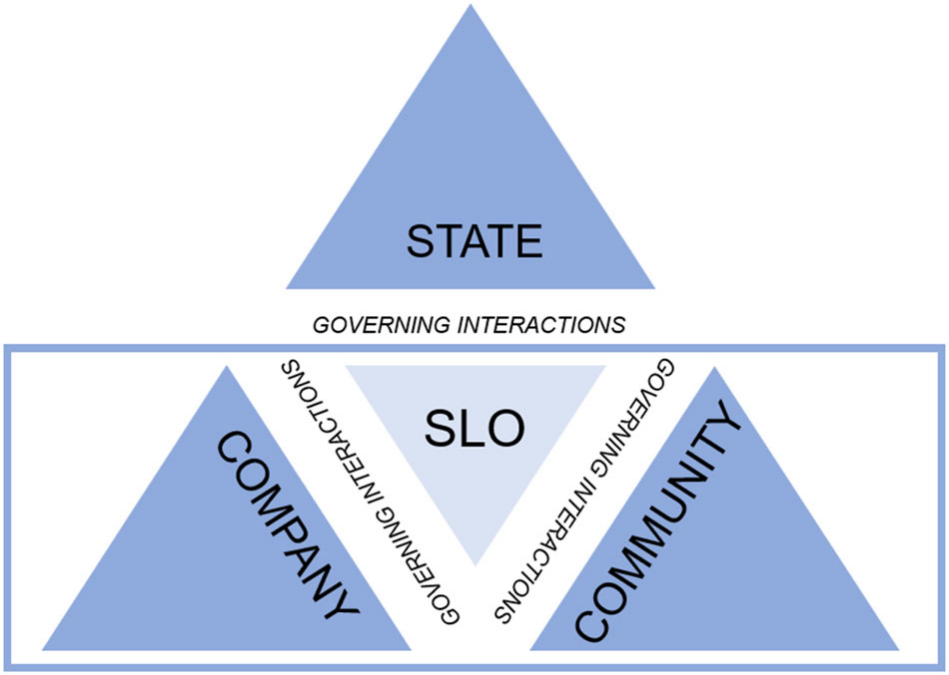
Tripartite SLO framework developed from Prno and Slocombe (2012) and Jentoft and Chuenpagdee (2015)

To investigate the approach used by mining companies within a governance arrangement, this study utilizes a theoretical framework based on the Prno and Slocombe’s model of the state, society, and market interaction in mining activities (2012) and the interactive governance theory from Jentoft and Chuenpagdee (2015) to assess difference corporate approaches. In this paper the focus is placed on the bottom section of the framework, specifically the company approach to community engagement (see Fig. 1). Using this integrated framework allows for the examination of both corporate policy and practice – an overview of corporate practice in action.

## Cases

Both cases are representative of established mining operations located in remote areas with cold climates and, most importantly, in proximity to Indigenous communities that continue to engage in traditional activities. The respective companies are currently operating mines without contestation from Indigenous communities, however the degree of collaboration differs. Understanding the different corporate approaches, and the context that provides space for collaboration, provides an important comparison for company-community relations and SLO.

### Cameco (Athabasca Basin)

The Athabasca Basin in northern Saskatchewan, Canada, is home to high-grade uranium reserves with ore grades up to 100 times the world average. Operations in the Athabasca Basin include the two highest grade mines in the world (Cigar Lake/McLean Lake and McArthur/Key Lake), as well as Rabbit Lake. These operations account for the entirety of uranium production from Canada over the last decade, which in 2019 was 13% of global supply (World Uranium Mining – World Nuclear Association (world-nuclear.org). McArthur River/Key Lake and Rabbit Lake were in safe states of care and maintenance from 2018 to the end of 2022, when production recommenced. Because of the sensitivity around uranium, particularly in terms of public perception, Cameco has devoted additional resources to building acceptance for their operations and regularly monitor the level of approval in the communities connected to their operations which sat at 78% in 2021 for northern Saskatchewan (Cameco 2021).

The Athabasca Basin sits within the Northern Administration District (NAD) of Saskatchewan, which accounts for almost half of the province’s territory at 268,390 square km but is home to only about 37,000 people in 45 communities. Most of this territory is state owned. The region is known for innovative IBAs with First Nations communities, including English River First Nation (ERFN). ERFN is composed of seven reserves: Cree Lake, Porter Island, Elak Dase, Knee Lake, Dipper Rapids, Wapachewunak, and LaPlonge. The main reserve is in Patuanak, near the Churchill River, 250 km northwest of Meadow Lake, Saskatchewan. ERFN is a Dene First Nation with about 1500 members. Like many northern Indigenous communities, the ERFN relies to a significant extent on land-based activities such as hunting and trapping for subsistence and income; but the wage economy and government transfers make up the two of three pillars of the local economy. The ERFN is a signatory to Treaty 10. Historic treaties are agreements made between the Crown and First Nations that define ongoing rights and obligations in exchange for land.

Cameco and ERFN maintain a strong working relationship, where discussions and decision-making are viewed as mutually beneficial and constructive (Poelzer et al. 2023). In other words, Cameco has established a strong SLO with ERFN which raises important questions on their historical and current practices that led to this outcome.

### Boliden (Gällivare)

Gällivare is a traditional mining town in northern Sweden with several operating mines located on traditional Indigenous (Sami) lands. One company operating in the municipality, Boliden, is extracting copper and gold in two open pit mines as part of its Aitik operations, while also planning a new mine in Liikavaara, and possibly another in Nautanen. All of these establishments are located within a 30 km radius from the Gällivare town center. Gällivare municipality is 16,818 square km, has a population of 17,529 persons, and hosts four Sami reindeer herding communities (RHCs), all practicing reindeer husbandry. Gällivare Sami RHC keeps their reindeers in the forest land around the town of Gällivare and is most affected by Boliden’s activities. In addition to practicing reindeer herding, the Sami RHC was part of starting up Ávki, a development company to support various kinds of Sami business and cultural projects. Gällivare has a mixed Sami and Swedish population (Swedish majority) and main land uses are mineral extraction, forestry, reindeer husbandry, tourism and hydro power production.

Copper was first found in the Aitik and Liikavaara area in the 1930s. The first mine in Aitik was in operation in 1968 with a production capacity of 2 Mton per year. Since then, the Aitik mine has been expanded several times, and Boliden now has plans to increase production capacity in Aitik to 45 Mton per year. Two projects have significant impacts on Sami reindeer herding in the area: AITIK 36 and the proposed Liikavaara project. Aitik 36 refers to Boliden’s project to expand the production capacity to 36 Mton per year. In 2006, Boliden submitted an application to the County Administrative Board (CAB), and a number of Court rulings between 2005 and 2009 granted Boliden permission to expand the operations. Formal consultations with Gällivare Sami RHC took place in 2006. As stated in the EIA, reindeer herding was expected to be affected by significant loss of grazing and calving lands; impacts on corrals used for marking and slaughter; blocked passages and increased workload. Mitigation measures included the construction of a passage across the new railway siding to enable reindeers to pass.

While Boliden operates without serious contestation, it has been noted that their relations with Gällivare Sami RHC show a lack of responsiveness and dissatisfaction regarding compensation (Beland Lindahl et al. 2023). This underscores an overall sense of an uneven distribution of benefits which leaves Boliden with a weak SLO, particularly when compared to Cameco.

## Methods

### Document and Interview Analysis

Research was conducted using a comparative case study approach (Yin 2009) involving methods consistent with primary (interviewing) and secondary (document review) qualitative methods to explore the relationship between government and Indigenous communities. To understand industry-community relations, this study looked that the corporate approach of two mining companies: Cameco and Boliden. The cases are both located in northern jurisdictions – a region rich in natural resources and the homelands to Indigenous populations. Both companies operate in countries that have relatively strong governing structures, a long history of governments interest and engagement on mining issues and are examples of cases with on-going dialog between companies and communities with no open contestation. This approach would also allow for an in-depth analysis of a specific subject area (Creswell 2014). These cases also have some differences in their governance context, historical development, minerals being extracted, and Indigenous rights.

Semi-structured interviews were conducted with company representatives from each of the cases to understand the current strategies used in their relationship with Indigenous communities, along with the historical development of community engagement. The equivalent to the manager of community engagement was interviewed from each company, along with a supplementary interview with another staff member from each company. A semi-structured interview approach allows for data comparison between cases while gathering important detail and nuance particular to each case (Turner 2010). The main objective of the interviews was to gain an understanding of the company perception of the effectiveness of their approach in developing relationships with Indigenous communities affected by their mining operation. Thus, the interviews were guided along the themes of corporate policy and practice, personal experience and approach, and perception of working with Indigenous communities. Due to data management and confidentiality rules, the names and precise titles of the interviewees are kept anonymous. All the interviews were coded and analyzed on NVivo.

The document analysis included publicly available materials for each mining company, including annual project reports, corporate sustainability reports, and previous academic research. Analysis focused on company statements and documentation of engagement with Indigenous communities, particularly partnerships related to economic and social development or environmental monitoring. Unfortunately, specific agreements between companies and Indigenous communities were not included in the analysis because those signed between Boliden and Gällivare SRHC remain confidential.

## Analysis of Corporate Approach

### Historical Context

#### Cameco

As uranium mining expanded in the north of the province of Saskatchewan, the Provincial Government established the first Mineral Surface Lease Agreement (MSLA) in 1978. This agreement arose in accordance with the Provincial Lands Act which provided a legislative framework that enabled producers to acquire rights to use surface land and granted landowners compensation. At public hearings in the late 1970s, northern Saskatchewan residents expressed interests in employment and business opportunities related to uranium mining in the area (Government of Saskatchewan 2018). This led to the provincial government and the local land users, primarily Indigenous peoples, to enter into an agreement to address these concerns – a framework for government to promote the sharing of benefits in broader areas. The MSLA ensures that mining companies engaged cooperatively with the communities and have measures in place to train, employ and provide opportunities to local community business (Parsons and Barsi 2001). Further, mine operators are expected to negotiate and enter into Human Resource Development Agreements (HRDA) for each mine site. The HRDA was introduced into the MSLA in 1989 and was signed between mining companies and the Government of Saskatchewan. This agreement focuses on “recruiting, hiring, training and job advancement opportunities for residents of Saskatchewan’s north and are signed by the proponent and the Ministry of the Economy” (Government of Saskatchewan 2014). These agreements have been signed by mining companies in northern Saskatchewan, leading to the mining sector becoming industry leaders in Indigenous employment and business procurement.

Cameco has an MSLA with the provincial government (Ministries of Environment and the former Ministry of First Nations and Metis Relations or Northern Affairs), which contains many provisions on "enhancing benefits to, mitigating impacts on, and engaging Indigenous communities" (Scott 2016). Therefore, all mining companies operating in the Northern Administration District (NAD) are required to make four northern commitments, which are employee services, education promotion, community vitality and public involvement, and report progress to the government (Parsons and Barsi 2001). However, follow-up and monitoring programs are focused heavily on biophysical effects monitoring in the local project environment, which is carried out by industry and regulated by the government. However, before a MSLA can be signed/entered into, an approval pursuant to The Environmental Assessment Act (Saskatchewan) must be granted.

The uranium mining and milling industry is the only mining industry in Canada that requires a federal licence to operate under federal legislation. The uranium industry is overseen through all stages of its lifecycle by an independent administrative tribunal, the Canadian Nuclear Safety Commission (CNSC). Beyond this federal licence to operate, mines and mills in Saskatchewan require an approval to operate from the Saskatchewan Ministry of Environment. Thus, a proponent in the uranium mining industry in Saskatchewan must meet regulatory requirements and permitting processes as set forth by both the federal and provincial governments.

#### Boliden

In Sweden, the process of establishing a mine is governed by the Minerals Act and the Environmental Code. Two government agencies coordinate the processes related to each legislation, the Mining Inspectorate and the County Administration Board (CAB) respectively. The first stage of the process falls under the Minerals Act which requires the project proponent to develop a work plan for exploration to receive an exploration permit from the Mining Inspectorate. The company must also share the development plan with the affected landowners and land users, including Sami RHC, who can then report to the Mining Inspectorate if they have any issues with the plan. If the exploration permit is granted, then the proponent can apply for a minerals concession if they decide the ore deposit is worth extracting. Concession applications also fall under the purview of the Mining Inspectorate and the Minerals Act serves as the guide. First, it involves an assessment of the economic viability of the project along with the company plans regarding health and safety. This includes conflict with existing land uses and compliance with the Environmental Code (Pettersson et al. 2015).

Because the Environmental Code is administered and interpreted at the county level in Sweden, each CAB is responsible for determining whether the project meets environmental standards. Importantly, if the proposed mining area impedes on reindeer herding then an assessment of this conflict must be undertaken as well. At this stage, consultations are recommended but are not a requirement of the Minerals Act. When these criteria are fulfilled, the Mining Inspectorate grants an exploitation concession. However, mining cannot begin exploitation without an environmental permit as required by the Environmental Code for environmentally hazardous activities.

Boliden first applied and were granted a Mining Concession for Liikavaara by the Mining Inspectorate in 1999. At the time, the Same RHC stated that they were in principle against a mine establishment in the area. Following new consultations in 2017 and 2018, Boliden applied for a Mining Concession to extract copper, silver gold and molybdenum in Liikavaara for a period of 25 years. The concession area is located within an area designated as a national interest for mineral extraction and reindeer husbandry, i.e. an assessment has to be made to what extent the two are compatible or which interest to prioritize. An EIA was prepared by Boliden’s consultant Enetjärn, including an impact assessment on reindeer husbandry based on nine consultation meetings with Gällivare Sami RHC. Impacts on reindeer husbandry, such as additional loss of grazing land, loss of key areas affecting the functionality of the entire territory and reindeer herding community, barriers for migration, possible health hazards for reindeers and additional disturbances by noise and traffic were identified. However, the analysis of the reindeer herders was only one out of several sources that influenced the consultant’s assessment. While the consultant (Enetjärn) concluded that the impacts are moderate or small (at least after closure and after treatment), the Sami RHC assessed them as uncertain and substantial, or major.

In 2019 the CAB recommended the Mining Inspectorate to deny the permit until impacts on the adjacent aquatic Natura 2000 areas have been tried according to the Environmental Code. Accordingly, Boliden applied to the Land and Environmental Court to change its existing environmental permit for Aitik to cover extraction in Liikavaara. The CAB asked Boliden to further clarify expected impacts on reindeer husbandry and mitigation measures; how reindeer husbandry, “as a public interest”, could be sufficiently cared for; and the conclusion that mining is compatible with reindeer husbandry in terms of the national interest. The Land and Environmental Court approved the EIA and granted an environmental permit For Liikavvara in April 2021. However, the Mining Concession is still under consideration. This plan for extraction highlights the cumulative effects of gradual expansions of several ongoing projects and permits. Previous expansions affected reindeer herding negatively as new land areas were taken into use for mining, infrastructure development and deposits; lakes were emptied and rivers rerouted; and already existing impacts such as noise, dust and emissions to air and water increased (Boliden 2006). The planned expansion in Liikavaara will involve additional land use change and this will require engagement with Sami RHCs to minimize the impacts.

### Corporate Policy and Agreements

#### Cameco

Since its creation in 1988, and especially after its initial public offering in 1991, Cameco has worked extensively with northern communities to build trust regarding their operations and corporate practices. With most of the population of northern Saskatchewan Indigenous, Cameco prioritized working with these communities - both for acceptance of uranium mining and to benefit from a local, stable workforce. After several years of discussion and negotiations, in May 2013, the English River First Nation and Cameco Corporation (along with AREVA Resources Canada Inc., another uranium mining company) announced the signing of a collaboration agreement that will strengthen the relationship between the parties and formalize how benefits from uranium mining will be shared with the ERFN community (Cameco Corp. 2015). The agreement provides a framework and guiding principles for long-term working relations. It sets out Cameco’s obligations to these communities under four main pillars: workforce development, business development, community investment and community engagement and environmental stewardship (Cameco Corp. 2015). In particular, the agreement specifies how the parties will work together, combining business conditions with customary demands, land rights and environmental management. The agreement clarifies how the ERFN will support the project if Cameco fulfils its advisory and partnership responsibilities as set out in the agreement.

This agreement builds on the historic relationship between Cameco and the ERFN around community development as well as the commercial relationship with community-owned businesses. Based on the existing Cameco and AREVA mining operations, the potential value of the agreement is estimated to be $600 million in economic benefit for the community over the next ten years. This amount could remarkably increase should projects like the proposed Millennium Mine come into operation in the future. The economic benefit will come in the shape of business contracts and employee wages. Moreover, the companies will also be responsible for signing bonuses, milestone payments and annual community investment (based on mine production for ERFN community development initiatives). Not only is this a business partnership, but it is also a form of social and economic development. Revenue generated from mining operations will help in the development of the ERFN youth, with funds directed towards education, health, and wellness.

The ERFN is currently represented through Des Nedhe Group, a business development company. Des Nedhe owns Tron Construction & Mining LP, a general contracting services company that specializes in construction projects and maintenance contracts. The company also engages in heavy earthmoving, electrical, mechanical, pipeline and environmental cleanup for the mining sector. Des Nedhe and Tron were incubated through procurement activities created from the relationships with Cameco and have diversified substantially to other sectors and projects, thus creating a variety of economic development opportunities for the nation and its members. Over the course of decades, the result of interaction between Cameco and ERFN is a mutually beneficial relationship that brings positive economic outcomes for both parties. Communication occurs regularly, especially with the recent volatility in uranium prices, but the result of the trust between company and community is on-going collaboration and partnerships.

#### Boliden

Boliden’s operations that use land, exploration, extraction, and rehabilitation, in the Gällivare area overlap with those of the Sami RHCs. One of the central mechanisms to managing the shared use of land in these areas is through private agreements. In general, these agreements are based around principles of cooperation, development and compensation and are negotiated between Boliden and the Sami RHCs. Because reindeer herders possess specific rights regarding land use related to reindeer husbandry, a central component of these agreements is based on the impacts to reindeer and compensation. However, the agreements between mining companies and Sami RHCs in Sweden remain private and the contents known only to the signatories.

In 2021 Boliden released a document (Boliden’s Indigenous People commitment) that describe their responsibility as a corporation to engage with communities with the goal of building trust. It outlines six points regarding their commitments on understanding the rights and interests of Indigenous Peoples and using agreed upon engagement practices that lead to consent, collaboration, and agreements. Over the years, Boliden has developed more regularized communication with Sami RHCs. The company maintains continuous dialog with the stakeholders and conduct several consultation processes each year, where Indigenous communities, along with the public and various business owners, are invited to attend and submit their views. Ensuring that the consultation process works well is essential for designing activities and projects in the best possible way and giving everyone the opportunity to express their views.

In Boliden’s annual reporting they reference their strategy is to act responsibly and to build trust with local stakeholders to get SLO to continue operations. They point to open dialog and cooperation with local communities, which enables the company to find solutions that are beneficial to both sides and mitigate negative consequences. Part of their policy is an understanding that different interest overlap, particularly around land use, but the company has been able to avoid significant disputes. In their efforts to reducing disputes, Boliden has entered into a number of research projects aimed reducing impacts. One development projects with Sami includes “Porokello,” a warning system for traffic to avoid accidents, used in Finland and at the Kevitsa mine. Boliden, the Sami villages in the Boliden Area, and the local contractor, Renfors Åkeri, have jointly agreed to test this system with the goal to reduce the number of accidents and improve safety for drivers, reindeer herders and reindeer. Another project involves the re-establishment of lichens. Because lichens are a staple food source for reindeer, its proliferation in the area is central to the future of reindeer husbandry. Pilot tests have been set up in Boliden and Aitik in partnership with the Swedish University of Agricultural Sciences.

### Company Perception

#### Cameco

Cameco also acknowledged that relationship building with Indigenous partners has developed significantly over time and much of that is premised on transparency. To establish a foundation of transparency and trust, the company points to the importance of ongoing and two-way communications about project performance and emerging issues and concerns. Cameco indicated that the way community concerns and interests were addressed helped the company gain the trust of Indigenous communities. Most central was the company being transparent in what its interests were concerning the land and opening a communication channel to know and understand the interests of the affected communities. One representative from Cameco stated:

*ERFN has a traditional territory, and they’re asserting their Aboriginal and Treaty Rights that need to be respected.*

This sentiment highlights the perspective Cameco holds on sharing land with Indigenous communities and understanding how working with communities that hold specific rights in central to their operational decision making. Cameco referenced that when looking at the environment and land use they engage with communities, create business-friendly relationships, and win-win solutions to allow us to access land and mitigate any risks. They also point out that signing agreements meant a recognition of Indigenous rights. The community relations manager pointed out:

*Engagement between Cameco and the communities under the CAs (community agreements) occurs primarily through an established process (community and industry representatives) that meets regularly to discuss operational and environment-related matters of importance to the communities… The agreement builds on the historic relationship as well as commercial relationship with businesses owned by the communities.*

The collaboration agreement between Cameco and ERFN was signed to ensure that the mining operation would continue to deliver economic benefits to ERFN communities; and an ERFN-led committee was established to oversee industry’s activities. Connecting the work of the mining company to important economic benefits through local jobs and income generation were central in stabilizing ongoing community-industry relationships. The collaboration agreement is viewed a product of respect, and a direct effect of Aboriginal and Treaty Rights. Importantly, these agreements not only set terms for business and environmental benchmarks, but also establish mutually agreed processes around engagement.

#### Boliden

Boliden and Gällivare Sami RHC have a long history of interaction. According to Boliden, consultations are continuous and ongoing with the affected Sami RHCs regarding exploration, operations, project development and rehabilitation, including preparation of the EIA reindeer analysis. Private agreements on cooperation, development and compensation are negotiated and, for existing mining operations, in place. At the early stages of negotiation, however, the argument from the Sami perspective was that the process was imbalanced, and Boliden was perceived as unresponsive. However, both parties agree that relations have developed and improved over time. The process leading up to Aitik 36 relied to a high extent on informal interaction and private agreements between the Sami RHC and Boliden. While both parties agreed that personal relations between the company and the reindeer herders have developed in a positive way, there are still questions regarding the quality and outcome of interaction. A goal in the future is for participation to be more strongly coupled with substantial influence in decision making. The interactions between the company and the Sami RHC are developing independently of state supervision or intervention.

While the mining activities in Aitik have generally expanded as Boliden planned, some mitigation measures have been taken, and the SRHC has adapted in part due to the compensation provided by the company. One of the underlying components to the relationship is the importance of mining to Gällivare municipality. While agreements have been reached regarding the current mining operations, it remains to be seen how agreements will emerge from the new mine establishment in Liikavaara. Regarding these new developments, the community relations manager stated:

*We are very focused to have established a good relation(ship) with the Sami in the area and make good working plans. Some of them (consultations) occur in good time to take their saying into consideration when we do things and how we do things and during project development. We have been hearing meetings and listened to them and that continues all the way through the permitting processes and also during operations.*

The description of the efforts made by the company offers insights into their relationship with the SRHC, as they are based primarily on consultation. Of particular importance is that their input is accounted for when it can “occur in good time.” This is a significant difference from the relationship between Cameco and ERFN where decision-making is done through a more systematic and coordinated approach.

One point of contention for both the mining company and the Sami RHCs is the overall governance of the mine establishment system. While Boliden is relatively satisfied with the performance of the permitting system, there have been delays, and they call for better coordination between different regulations and authorities. An example is coordination regarding infrastructure development:

*If you are supposed to build one (ecoduct) over a big road like E10, it’s really costly and it’s actually Trafikverket (Transportation Agency) that has responsibility to do that, in our opinion. And there is already work regarding that they have started up. They have had hearings. We hear Sami from the coast up too and saw that there are proposals on different places to actually build them.*

Issues around construction of the mine, especially new roads, have placed both Boliden and the Sami RHCs in a difficult negotiation position where determining whether the company or state should be responsible for costs is unclear Table 1.

**Table 1:** Comparison of corporate approach

	Cameco	Boliden
Context	Strong SLO - Long history of engagement with Indigenous communities, under the legislative framework that includes Treaties	Weak SLO - Engagement with Indigenous communities as stakeholders with land use rights
Corporate Policy	Develop comprehensive agreements with communities that include economic and social development; joint environmental monitoring	Consultation regarding operations, particularly mine expansion and new projects; establishing research projects with SRHC partners
Company Perception	Interdependence between company and community, corporate culture that recognizes Indigenous communities as partners	Building understanding of SRHC needs and finding practical solutions to mitigate impacts on reindeer

## Discussion

Social license to operate is synonymous with company-community relationships in the minerals sector. These relationships are particularly sensitive when considering Indigenous communities as finding ways to share land for industrial and traditional activities become complicated. However, this also opens the opportunity for companies to find innovative solutions, outside the purview of government, that satisfy the needs of the community while maintaining profitable operations.

The Cameco case of engagement with English River First Nation exemplified a well-developed collaboration and partnership. Critically, it appears to be highly valued by the company. The perception of interaction is generally assessed as high trust and collaborative, with no major disputes or legal litigation raised. Most importantly, regarding corporate engagement, is that the quality of interaction has led to formalized agreements that institutionalize the involvement of Indigenous corporate actors (Indigenous-led businesses), different forms of partnerships or formalized co-management structures, and a non-hierarchical degree of influence. In this case, there is a clear governance arrangement that features little government involvement. Because Cameco and ERFN have established both procedural and outcome-based protocol related to mining operations, the company has developed a high level of trust with the community and a strong SLO.

For Boliden, the nature of company-community relationships has improved over time, particularly in the past few years. The interactions between the company and Sami RHCs have become more routinized, both through formal and informal channels. And while the degree of dialog has increased, the degree of company-community collaboration is significantly less than that between Cameco and ERFN. While personal relations between the company and SRHC were mutually appreciated, the Indigenous community still lacks substantial influence of decision-making. While the agreements between Cameco and ERFN contain provisions similar to a co-management governance arrangement, the agreements between Boliden and Gällivare Sami RHC are oriented more towards compensation. This can be, in part, due to the lack of Indigenous self-government in Sweden. Further, partnerships between the company and Indigenous communities are limited to research projects that look at the impacts on reindeer herding but do not offer the same guarantees as the comprehensive agreements between Cameco and ERFN. Decision around issues related to infrastructure development were sometimes differ to government agencies to manage, which indicates that governance is still strongly linked to the state. The combination of softer partnerships with Indigenous communities and turning to the state to solve disputes demonstrate why the company holds a weak SLO.

## Conclusion

While both Cameco and Boliden experience little to no disruption to corporate activity due to disagreements or contestation from ERFN and Gällivare Sami RHC, respectively, the scope and scale of collaboration between the company and Indigenous community differ significantly. Cameco currently engages ERFN as a partner in its corporate practices while Boliden’s approach is akin on stakeholder relations. As a result, the purview of the agreements and collaboration between Cameco and ENFN are towards future development, incorporating social, economic, and environmental factors. While Boliden is moving forward in terms of its recognition of Indigenous values and interests, much of the current collaboration is piecemeal, rather than a broad integration. This raises an important issue regarding SLO, where companies need to find the best opportunities to work within the legislative framework to develop partnerships with Indigenous communities. In cases where companies continue to lean on the state and its legislative requirements, the possibility for achieving a strong SLO is limited.
